# Reassigning an amber codon to selenocysteine using a functional cysteine suppression pair in *Escherichia coli*

**DOI:** 10.1016/j.synbio.2026.06.011

**Published:** 2026-07-29

**Authors:** Shaopeng Niu, Jingyan Wei, Mengna Li, Borui Guo, Qi Yan, Yu Huang, Zhenyan Jiang, Jian Song

**Affiliations:** a School of Pharmaceutical Science, Jilin University, Changchun, 130021, PR China; b School of Microelectronics, Shanghai University, Shanghai, 201800, PR China; c Key Laboratory for Molecular Enzymology and Engineering of the Ministry of Education, Jilin University, Changchun, 130000, PR China

**Keywords:** Selenoprotein, Glutathione peroxidase, Genetic code expansion, Amber suppression

## Abstract

Selenoproteins, characterized by the presence of selenocysteine (Sec) residues, are widely distributed across all domains of life. The unique attributes of selenoproteins are conferred by Sec, which is genetically encoded into nascent polypeptide chains through the recoding of the UGA codon. Human glutathione peroxidase 1 (GPx1) is a selenoprotein, which plays a crucial role in maintaining redox homeostasis. However, its complex recoding mechanism presents a major challenge for efficient selenoprotein expression in *Escherichia coli*. In this study, we engineered a functional amber suppression system that consists of a cysteinyl-tRNA synthetase variant (M38-18) and its cognate tRNA^Cys^ variant (M1-1). These components were developed through directed evolution of the native *E. coli* pair. Using this system in *E. coli* C321.ΔA.exp, we successfully achieved recombinant expression of GPx1 and a GPx1 mutant, GPx1-C-S-49TAG featuring serine substitutions for all cysteine residues, with enzymatic activities of around 420 U/mg and 290 U/mg, respectively. This work provides an alternative platform for selenoprotein production in bacteria and establishes an innovative strategy for the scalable biosynthesis of GPx and related therapeutics.

## Graphical abstract

**Figure:**
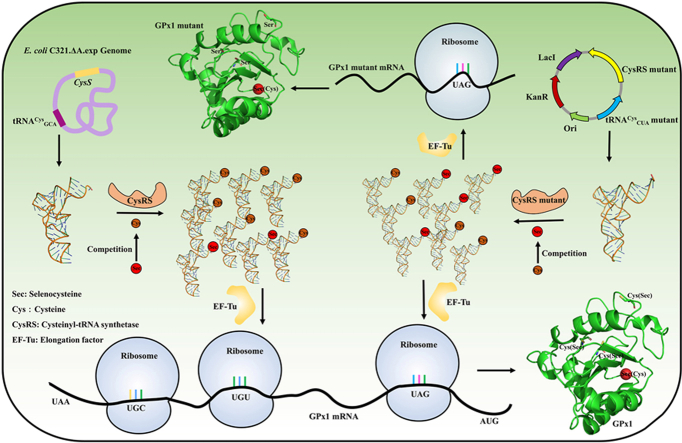

## 1 Introduction

Selenocysteine (Sec), recognized as the twenty-first amino acid, is genetically incorporated into selenoproteins through a unique recoding mechanism. Its distinct chemical properties enable selenoproteins to perform essential biochemical and biophysical functions in all three domains of life [1]. In humans, twenty-five selenoproteins have been identified to date [2]. Among these, glutathione peroxidases (GPx1–4 and GPx6), which have selenocysteines at their active sites, play a pivotal role in oxidative defense and in maintaining redox homeostasis [3]. Oxidative stress has been linked to a variety of physiological and pathological conditions, including aging, cancer, neurodegenerative disorders, and cardiovascular diseases [4,5]. As a result, the study and production of glutathione peroxidases are drawing increasing attention, particularly for their potential applications in therapeutics.

Site-specific incorporation of Sec is mediated by redefining the UGA codon, which is typically recognized as a stop codon. To achieve this, living organisms have evolved an exclusive Sec-specific elongation factor (SelB in prokaryotes or eEFSec in eukaryotes), a conserved mRNA motif known as the selenocysteine insertion sequence (SECIS), and a distinguishing Sec-specific tRNA^Sec^. In prokaryotes, tRNA^Sec^ is initially recognized and aminoacylated by endogenous seryl-tRNA synthetase (SerRS) to form Ser-tRNA^Sec^. This intermediate is then converted into Sec-tRNA^Sec^ by selenocysteine synthase (SelA), with selenophosphate produced by selenophosphate synthase (SelD) denoted as selenodonor [6]. When the translating ribosome encounters a UGA codon followed immediately by a SECIS element, SelB, in conjunction with SECIS, recruits and delivers Sec-tRNA^Sec^ to the ribosomal A site to facilitate the reassignment of a stop codon in mRNA to a sense codon encoding selenocysteine [7].

With detailed knowledge of Sec biosynthesis and its incorporation mechanism, it can be expected that the expression of human selenoproteins, including GPx, in *E*. *coli* is a challenging task due to the potential need to reconfigure the SECIS element. A recent effort by Anna Thaenert aimed to fuse the 16S rRNA of an orthogonal ribosome with SECIS to develop an mRNA–ribosome complex called RiboU and demonstrated its practicality for inserting Sec into the growing polypeptide chain at the appropriate site [8]. Furthermore, to circumvent the employment of SECIS and SelB, some chimeric tRNAs such as tRNA^UTu^, tRNA^SecUx^, and allo-tRNA^UTu2D^ have been innovatively proposed to implement site-directed incorporation of Sec [[9], [10], [11]]. Subsequent optimization yields tRNA^UTuX^ and tRNA^UTuT6^ for the purpose of enhancement of Sec insertion level [12,13]. These hybrid tRNAs can be recognized by the natural elongation factor (EF-Tu), just like canonical tRNAs, and exert their function of decoding the amber codon. These EF-Tu-mediated translation systems independent of SECIS have greatly expanded our capacity to produce selenoproteins.

Recent advances in genetic code expansion (GCE) have established in vivo methods in *E. coli*. The toolkit for GCE is composed of orthogonal pairs consisting of an orthogonal tRNA, which can't be charged by endogenous aminoacyl-tRNA synthetases (aaRSs), and an orthogonal aaRS that aminoacylates the orthogonal tRNA with an unnatural amino acid but doesn't charge endogenous tRNAs [14,15]. This system enables the incorporation of unnatural amino acids at predesigned sites, mainly at nonsense codons and quadruplet codons. As an example of selenoprotein production, Adarshi P. provides an approach to insert a photocaged selenocysteine in response to an amber codon using an orthogonal pair of tRNA^Ply^_CUA_ and Mm PCC2RS [16]. The caging group can be released upon UV irradiation. What's more, the amber codon is genetically recoded to incorporate Se-allyl selenocysteine (ASec) using an evolved tRNA^Pyl^-ASecRS [17]. Through palladium-mediated cleavage under mild conditions, ASec can be selectively transformed to Sec. Although these methods have given us a lot of inspiration, the caged group may bring about unpredictable impacts on protein activity and structure and a limited yield of selenoprotein.

It is widely recognized that Sec serves as a natural substrate of cysteinyl-tRNA synthetase (CysRS) [[18], [19], [20]]. In previous work by our group, the cysteine (Cys) codon could be residue-specifically replaced by Sec using a Cys auxotrophic strain for the expression of a GPx1 mutant [21]. However, this methodology is both resource-intensive and protracted due to the necessity of synthetic media and bacterial washing procedures. Furthermore, the expression of the GPx1 mutant might present limitations for investigations into protein characteristics [21]. In this work, we endeavored to leverage CysRS and its corresponding tRNA^Cys^ derived from *E. coli* to establish an efficient amber suppression pair as orthogonal as possible. After multiple rounds of screening, we identified a highly active pair, which we named M1-1 (tRNA^Cys^ mutant 1-1) and M38-18 (CysRS mutant 38-18). We then investigated its capacity to directly decode an amber codon to Sec in amber-less *E. coli* C321.ΔA.exp, a strain previously reported to have all 321 endogenous UAG codons replaced by UAA and its UAG-specific release factor 1 deleted [22]. Surprisingly, using the evolved pair of M1-1 and M38-18, recombinant expression of GPx1 was achieved with high activity even without altering the amino acid-binding domain of CysRS. Additionally, this pair was proven to be effective in expressing a range of other functional selenoproteins, including GPx2−4.

## 2 Materials and methods

### 2.1 Strains, plasmids and reagents

*E. coli* strain DH5α was used for plasmid propagation. The amber-less *E. coli* strain C321.ΔA.exp was obtained from Addgene (49018) and used for protein expression. The pGFIB and pRSF-DuetI vectors were generous gifts from Dieter Söll. The pACYC-184 and pCDF-DuetI vectors were obtained from Sangon Biotech (Shanghai, China). NADPH, GSH, IPTG, X-gal, and glutathione reductase (GR) were purchased from Sigma-Aldrich (St. Louis, MO, USA). Chloramphenicol, ampicillin, kanamycin, and spectinomycin were acquired from Sangon Biotech (Shanghai, China). All other chemicals were bought from Beijing Chemical Factory (Beijing, China) and were of analytical grade.

### 2.2 Plasmids construction

**Fig. 1:**
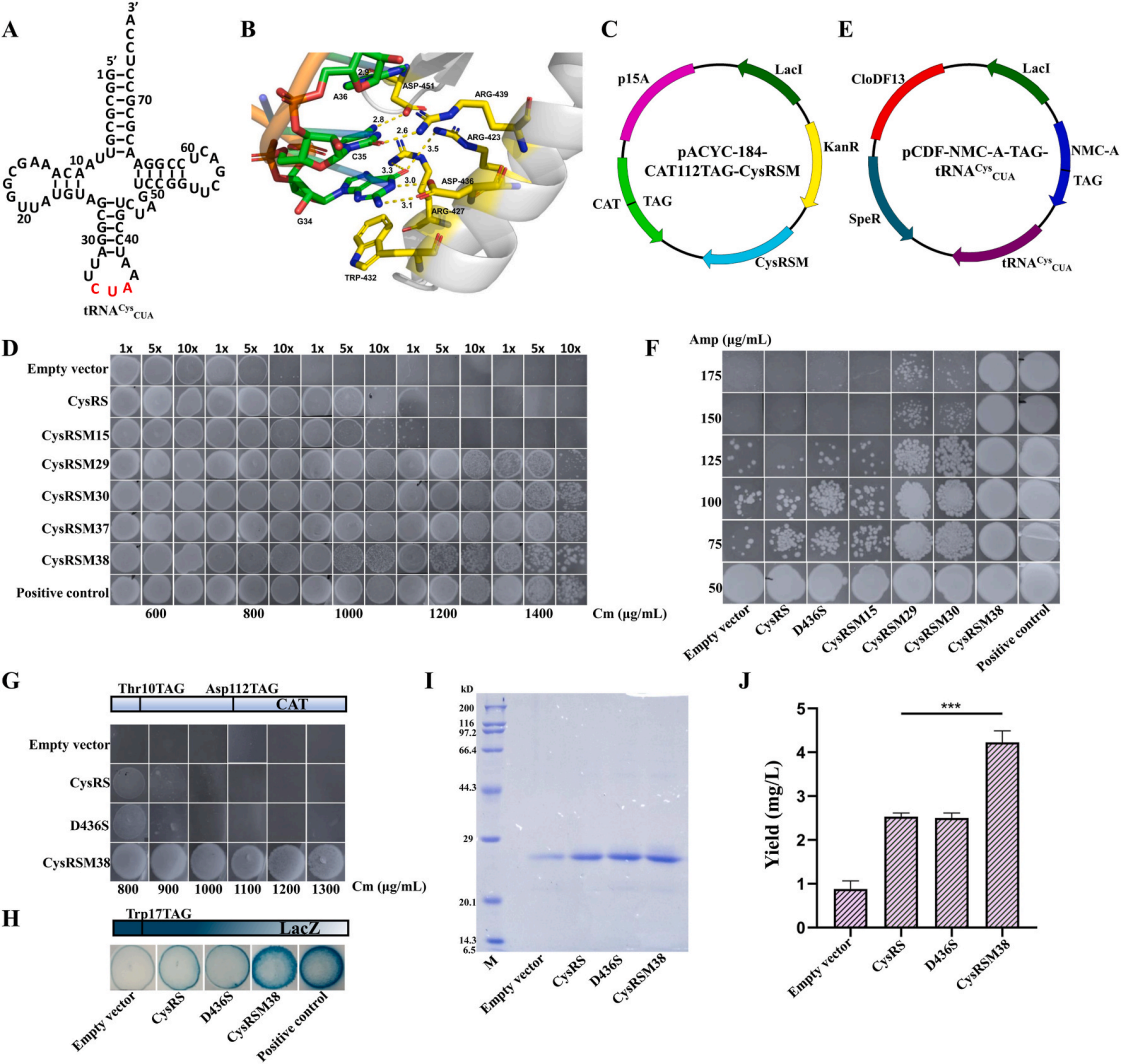
Evolution of the anticodon binding domain of CysRS. (**A**) Cloverleaf structure of *E. coli* tRNA^Cys^_CUA_, with the anticodon highlighted in red. (**B**) Structural representation of interaction sites within the anticodon domain of CysRS, visualized using the open-source PyMOL software based on the Protein Data Bank (PDB) entry 1U0B. (**C**) Schematic diagram of the plasmid used for chloramphenicol resistance screening. (**D**) Spot plate verification of chloramphenicol resistance of selected variants. (**E**) Map of the plasmid of NMC-A-TAG reporter. (**F**) Assessment of ampicillin resistance of different variants. Sequencing analysis confirmed that CysRSM37 and CysRSM30 are identical (Arg427Ala, Trp432Ile, Asp436Gly, Arg439Thr); consequently, CysRSM37 was excluded from the ampicillin resistance validation and replaced with CysRSM30. (**G**) Spot plate assay verifying chloramphenicol resistance using a CAT10/112TAG reporter. (**H**) Determination of β-galactosidase activity in control groups and M38. An amber codon was inserted at the 17th amino acid position of β-galactosidase. (**I-J**) SDS-PAGE analysis and quantification of GPx1 expression. CysRSM: CysRS mutant; D436S: CysRS Asp436Ser; Cm: Chloramphenicol; Amp: Ampicillin; M: Marker; Empty vector: pACYC-184-CAT112TAG; Positive control: No amber codons within reporters. Data represent the mean ± standard deviation (SD) (n = 3); ***P < 0.001.

The sequences of tRNA^Cys^ and CysRS were amplified from the *E. coli* genome (Gene IDs: 946414 and 946969) and cloned into pGFIB and pACYC-184 vectors, respectively. Site-directed mutagenesis and random saturation mutagenesis were conducted to generate pGFIB-tRNA^Cys^_CUA_ and pACYC-184-CAT112TAG-CysRS mutant (CysRSM). NMC-A β-lactamase (NMC-A) containing a TAG was synthesized by Sangon and cloned into pCDF-DuetI. During ampicillin screening, tRNA^Cys^_CUA_ was transferred into pCDF to produce pCDF-NMC-A-TAG-tRNA^Cys^. Protein expression in Fig. 1 utilized pColdI-GPx1-tRNA^Cys^_CUA_ and either pACYC-184-CAT112TAG-CysRS/CysRS variant or the pACYC-184 empty vector. The LeuQ promoter was obtained from the *E. coli* genome and subcloned into the *Xba*I site of pRSF-DuetI, followed by tRNA^Cys^_CUA_. Libraries of amino acid residues near the acceptor arm of M1-1, a more orthogonal variant of tRNA^Cys^_CUA_, were ligated into the *Nde*I/*Xho*I site of pRSF-M1-1. The plasmids pCDF-EGFP-TAG and pCDF-GPx1, along with pRSF-M1-1-M38-18, were used for fluorescence assays and selenoprotein expression, respectively. All constructed plasmids were validated by sequencing at Azenta Company. Primary primer sequences used in this study are listed in Table S1, including key primers for library construction and protein expression.

### 2.3 Screening and spot scheme

The plasmid of pACYC-184-CAT112TAG-CysRSM was transformed into *E. coli* C321.ΔA.exp-pGFIB-tRNA^Cys^_CUA_. The efficiency of library conversion was assessed by counting the number of colonies on an agar plate. After appropriate cultivation of the scraped bacterial suspension, the bacterial suspension was spread onto agar plates containing a gradient of chloramphenicol concentrations. Colonies from plates with higher antibiotic concentrations were collected and subjected to another round of spread plate screening. Selected clones from high-concentration plates were inoculated into 2 mL of Luria-Bertani (LB) medium containing kanamycin (50 μg/mL) and ampicillin (100 μg/mL) and cultured overnight with shaking. The OD_600_ values were then normalized, and the cell suspensions were diluted to various concentrations. Aliquots were spotted onto chloramphenicol plates with different concentrations. Superior colonies were selected for plasmid extraction and retransformed to confirm the results. Screening CysRS variants with higher affinity for M1-1 using ampicillin resistance followed a similar procedure apart from the different plasmids used.

### 2.4 Galactosidase and fluorescence assay

The plasmid of pACYC-CysRS/CysRS variant was co-transformed into *E. coli* C321.ΔA.exp along with pColdI-LacZ-TAG-tRNA^Cys^_CUA_. The resulting single colony was individually inoculated into 2 mL of LB medium with ampicillin (100 μg/mL) and kanamycin (50 μg/mL) and incubated overnight until the stationary phase was reached. After normalizing the OD_600_ of the bacterial cultures, 3–5 μL of each culture was pipetted onto pre-prepared LB agar plates containing 1 mM IPTG and 0.2 mg/mL X-gal. Plates were incubated at 37°C, and the colony color was observed.

EGFP was expressed from pCDF-EGFPTAG in the *E. coli* C321.ΔA.exp, which had been co-transformed with the plasmids pRSF-M1-1-CysRS/CysRS variant. A single colony was picked from the plates, and cells were grown at 37°C in 2 mL of LB selective medium supplemented with kanamycin (50 μg/mL) and spectinomycin (90 μg/mL) on a shaker until saturation was reached. Then, 3–5 μL of the normalized culture was spotted onto agar plates containing IPTG. IPTG was added to the remaining culture to a final concentration of 100 μM. Cultivation continued for 9–12 h, after which cells were harvested by centrifugation. The bacterial pellet was washed twice with phosphate buffered saline (PBS), resuspended in PBS, and the fluorescence intensity of the bacterial suspension was measured using a microplate reader (BioTek Synergy HTX) and normalized to optical density at 600 nm.

### 2.5 Protein expression, purification, and activity measurement

GPx was subcloned into pCDF-DuetI downstream of the CspA promoter, which needed low temperature to induce expression. The resulting recombinant plasmids were co-transformed into the *E. coli* C321.ΔA.exp along with pRSF-M1-1-M38-18. Starting from a single colony after transformation, cells were grown at 37°C in 2 mL of LB medium supplemented with kanamycin (50 μg/mL) and spectinomycin (90 μg/mL) in a shaking incubator. Subsequently, 200 μL of the overnight culture was inoculated into 200 mL of LB medium containing the same antibiotics and incubated at 37°C overnight. When the OD_600_ of the culture reached 1.2−1.5, the temperature was lowered to 20°C. After 4 h, protein expression was induced by adding 100 μM IPTG and 10 μg/mL Sec, followed by continued incubation at 20°C for 18 h.

Cells were collected by centrifugation at 5000× *g* for 5 min at 20°C, and the cell pellet was resuspended in 10 mL of lysis buffer. After sonication lysis and centrifugation at 20,000× *g* for 10 min, proteins were purified from the supernatant using an immobilized metal-affinity chromatography purification system with Ni–NTA resin. Following gradient elution with a low-concentration imidazole buffer, target proteins were eluted with a buffer containing 50 mM sodium phosphate, 300 mM NaCl, and 300 mM imidazole.

GPx activity was assayed according to the method described previously [23]. Samples were mixed with 50 mM PBS (pH 7.4), 1 mM EDTA, 0.25 mM NADPH, and 1 U of glutathione reductase (GR) in a cuvette and incubated at 37°C for 3 min. The reaction was initiated by the addition of 500 μM H_2_O_2_. GPx activity was determined by measuring the decrease in NADPH absorbance at 340 nm over time. Activity units (U) were defined as the amount of enzyme oxidizing 1 μmol of NADPH per minute at 37°C. Specific activity is expressed as U/mg protein.

### 2.6 Sodium dodecyl sulfate–polyacrylamide gel electrophoresis (SDS-PAGE) and western blotting (WB) analysis

After boiling in the SDS loading buffer for 10 min, purified proteins were subjected to SDS-PAGE, followed by staining with Coomassie brilliant blue R-250. For Western blotting, samples were electro-transferred onto nitrocellulose membranes. The membranes were blocked with 5% nonfat dry milk. Mouse monoclonal anti-6×His IgG was incubated with the membranes overnight. After washing three times with TBST, goat anti-mouse IgG conjugated to horseradish peroxidase was used to further incubate. Protein bands were visualized using 3,3′-diaminobenzidine (DAB) or a chemiluminescence developer following membrane washing.

### 2.7 Mass spectrometry analysis

Protein samples were desalted by ultrafiltration and dissolved in pure water. Electrospray ionization mass spectrometry of the protein product was acquired on the Orbitrap Exploris 480. The obtained mass data were processed via Xcalibur software. Theoretical mass values were obtained using an online tool (https://web.expasy.org/peptide_mass/) and subsequently subjected to manual adjustments to remove N-terminal methionine. For liquid chromatography-tandem mass spectrometry (LC-MS/MS) analysis, the samples were excised from the SDS gel, and the pretreatment steps were carried out as previously described [12,13]. Each sample was resuspended in buffer A (2% acetonitrile (ACN), 0.1% formic acid (FA)) and centrifuged at 20,000× *g*. Then, 10 μL of the supernatant was loaded onto an Acclaim PepMap C18 reversed-phase column (75 μm × 2 cm, 3 μm, 100 Å; Thermo Fisher Scientific, USA) and separated using a reversed-phase C18 column (75 μm × 10 cm, 5 μm, 300 Å; Agela Technologies, China) mounted on a Dionex Ultimate 3000 nano LC system. Peptides were eluted using a gradient and analyzed with a Q Exactive mass spectrometer (Thermo Fisher Scientific, USA). The eluates were directly introduced into the Q Exactive mass spectrometer, operated in positive ion mode and data-dependent acquisition mode, with a full MS scan range of 350 to 2000 *m*/*z*, a full scan resolution of 70,000, and an MS/MS scan resolution of 17,500. MS/MS scans were performed with a minimum signal threshold of 1 × 10^5^ and an isolation width of 2 Da. The MS/MS acquisition mode was higher-energy collisional dissociation (HCD), with normalized collision energy (NCE) systematically optimized at 28% and stepped at 20%. Raw MS/MS data were converted into MGF format using Proteome Discoverer 1.4 (Thermo Fisher Scientific, Waltham, MA, USA). Peptide identification was performed using Mascot software (version 2.3.01, Matrix Science, UK).

### 2.8 Statistical analysis

All parameters and calculated variables were analyzed for differences between the experimental group and corresponding controls using the Student's *t*-test implemented in GraphPad Prism 8. Differences were considered significant when the *P* value was less than 0.05 (indicated by an asterisk [*]). The letter n represents the number of biological replicates.

## 3 Results

### 3.1 Creation of an amber suppression variant of tRNA^Cys^ and preliminary evolution of CysRS

According to the DNA sequence of *E. coli* tRNA^Cys^ from the GenBank database, we engineered an amber suppression variant as illustrated in Fig. 1A. This variant was constructed via primer-directed synthesis and subsequently cloned into the pGFIB plasmid, regulated by the Lpp promoter. It is widely proposed that the anticodon and nucleotide U73 of tRNA^Cys^ are critical recognition elements for CysRS [24]. We hypothesized that the altered anticodon might result in a relatively orthogonal tRNA that could not be recognized by endogenous CysRS; therefore, we first evolved the anticodon binding domain of CysRS to obtain a variant with higher affinity for the tRNA^Cys^_CUA_. The anticodon binding domain of CysRS and its ligand, tRNA^Cys^, are depicted in Fig. 1B, from which we can clearly understand the patterns of interaction between them. We acquired the sequence of CysRS from the *E. coli* genome, and site-saturation mutations were engineered at amino acid positions 427/432/436/439. The resultant CysRS library was ligated into the pACYC184 vector, which had been pre-modified with an amber mutation at position 112 within the chloramphenicol acetyltransferase (CAT) reporter gene (Fig. 1C). An increase in chloramphenicol resistance would signify enhanced affinity of a mutated CysRS for tRNA^Cys^_CUA_. Following iterative rounds of initial selection, several advantageous mutants were identified, as depicted in Fig. 1D.

To identify the optimal mutant, we employed NMC-A β-lactamase (NMC-A) containing an amber mutation as a surrogate reporter system (Fig. 1E). The resulting ampicillin resistance profile, presented in Fig. 1F, highlights the superior performance of CysRSM38, abbreviated as M38 (Arg427Thr, Trp432His, Asp436Arg, Arg439Ser). For further validation, double TAG codons were introduced into the CAT open reading frame. Chloramphenicol resistance testing (Fig. 1G) indicated that M38 exhibited a marked improvement compared to the wild-type CysRS and Asp436Ser (D436S) mutant, which was reported to enhance the in vitro TAG suppression efficiency of tRNA^Cys^_CUA_ [25]. Color observations from Fig. 1H further supported this conclusion with β-galactosidase (*LacZ*) serving as the reporter. Furthermore, when evaluating the expression yield of the GPx1 protein utilizing the pColdI-GPx1-tRNA^Cys^_CUA_ vector, M38 also demonstrated greater effectiveness, with a yield approximately 1.5 times that of the CysRS. Fig. 1I and J detailed the SDS-PAGE analysis and quantification of yields for the M38 and control groups, respectively.

### 3.2 Construction of a more orthogonal tRNA^Cys^_CUA_ variant

**Fig. 2:**
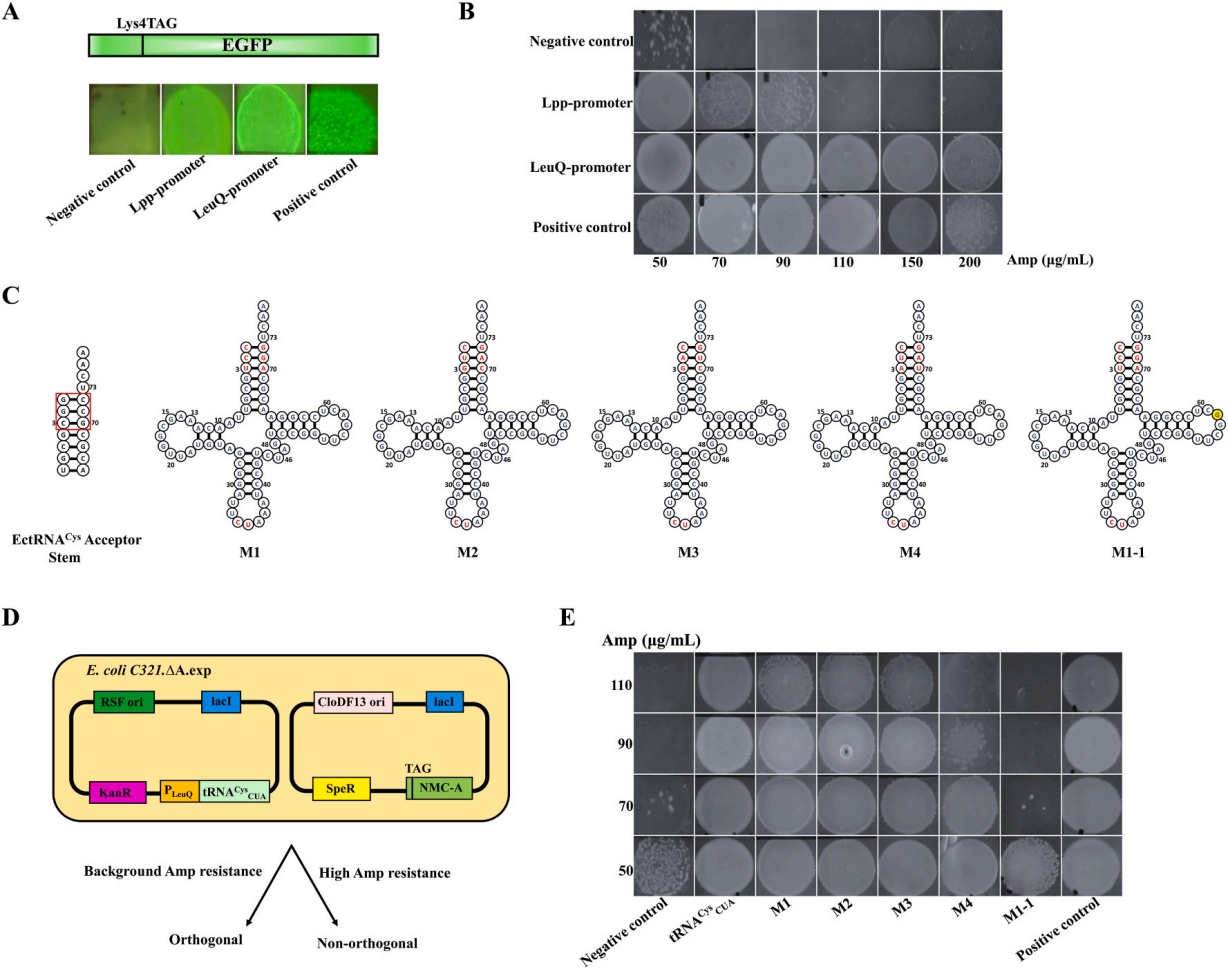
Design and characterization of a more orthogonal tRNA^Cys^_CUA_ mutant. (**A**) Fluorescence analysis to determine the function and strength of LeuQ promoter-mediated tRNA^Cys^_CUA_ transcription. (**B**) Comparison of transcriptional activity of the Lpp promoter and the LeuQ promoter using an ampicillin resistance assay. (**C**) Acceptor stem of tRNA^Cys^ (red frame) and cloverleaf structures of several variants engineered. Compared to the native tRNA^Cys^, mutations in the anticodon and the first three base pairs of the acceptor arm are highlighted in red. The additional A58G mutation in M1-1 is marked in yellow. (**D**) A schematic illustrating the use of the NMC-A reporter gene to determine orthogonality of distinct variants. A reduced level of ampicillin resistance is associated with diminished TAG read-through efficiency and lower cross-reactivity between the tRNA^Cys^_CUA_ variant and endogenous CysRS. (**E**) Analysis of ampicillin resistance in different tRNA^Cys^_CUA_ variants. Spotting assays were conducted using a bacterial culture at a normalized OD_600_ of 1. P_LeuQ_: LeuQ promoter. “Negative control” refers to the absence of any tRNA, and “positive control” is annotated as the lack of amber codons within reporters.

Despite the anticodon's alteration to CUA, it seemed that endogenous CysRS appeared capable of charging the tRNA^Cys^_CUA_ with Cys [26], as evidenced by the empty vector controls and CysRS controls in Fig. 1D and I. To mitigate potential non-orthogonality, we proceeded with a rational design and mutational approach targeting the acceptor stem to select for more orthogonal tRNA^Cys^_CUA_ variants. It is well-established that modifying the tRNA acceptor stem, which may harbor primary or secondary identity determinants for aaRS, is a standard and effective strategy for enhancing orthogonality in GCE [27]. Given that stronger promoters can amplify cross-reactivity and assist in identifying more orthogonal variants, we subcloned the tRNA^Cys^_CUA_ sequence from the antecedent pGFIB vector into the pRSF-DuetI vector and aimed to replace the promoter with the LeuQ promoter, reported to exhibit superior strength compared to the Lpp promoter [28]. Detection of fluorescence intensity in Fig. 2A revealed that the LeuQ promoter was successfully amplified from the *E. coli* genome and demonstrated greater efficacy than the Lpp promoter in suppressing the amber codon at a permissive site within EGFP. To further validate this result, we re-examined this promoter using the NMC-A-TAG reporter gene. Compared to the Lpp promoter, the LeuQ promoter conferred higher ampicillin resistance to tRNA^Cys^_CUA_, with a concentration difference of at least 100 μg/mL (Fig. 2B), further demonstrating that the LeuQ promoter exhibited a higher transcription efficiency, consistent with the conclusions drawn from the fluorescence intensity analysis described above.

Building on the stronger promoter LeuQ, we developed several tRNA variants, primarily modifying the initial three base pairs of the tRNA^Cys^_CUA_. Mutant 1−Mutant 4 (M1–M4) were generated through base pair inversions and substitutions, and an additional variant, M1-1, was also serendipitously generated through PCR during plasmid construction (Fig. 2C). As shown in Fig. 2D, lower ampicillin resistance correlates with reduced cross-reactivity with CysRS, while variants exhibiting background levels are orthogonal variants. Evaluation of ampicillin resistance showed reduced resistance levels for constructs M1 through M4. Relative to the parent tRNA^Cys^_CUA_, a concentration difference of at least 50 μg/mL was measured at an OD_600_ of 1. This disparity was further amplified in 10-fold diluted samples (Fig. S1). Surprisingly, variant M1-1 exhibited the lowest level of ampicillin resistance among all constructed variants, appearing almost indistinguishable from the background level (Fig. 2E). This indicated that the M1-1 variant featured sufficiently low cross-reactivity with endogenous CysRS to serve as a more orthogonal tRNA. This finding was further supported by the outcome of subsequent investigations using other reporters such as EGFP and GPx1.

### 3.3 Screening for CysRS variants with enhanced catalytic efficiency against the M1-1 variant

**Fig. 3:**
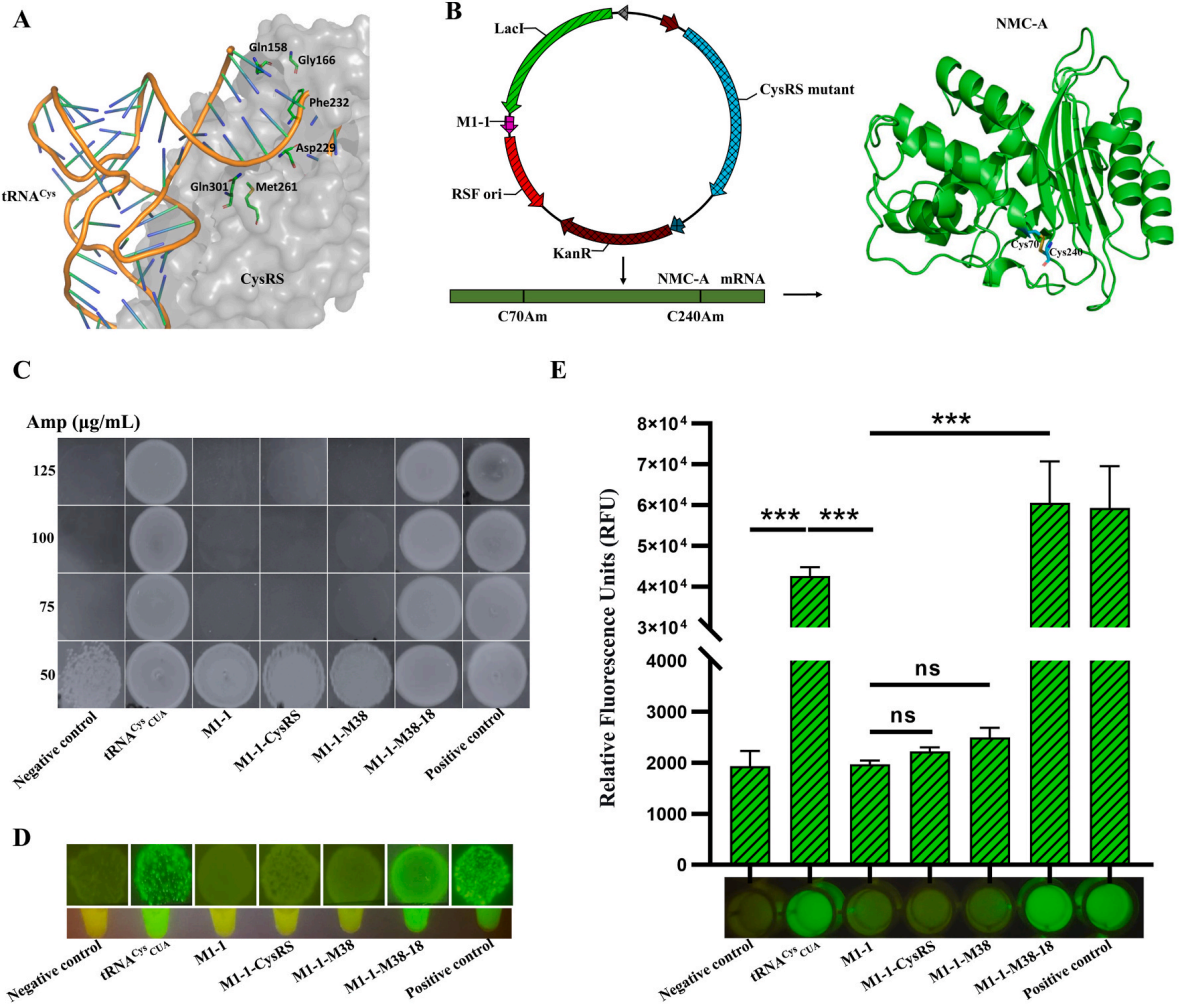
Screening CysRS variants with higher affinity for M1-1. (**A**) Structural illustration of selected residues surrounding the first three base pairs of tRNA^Cys^. Structural visualization of CysRS⋅tRNA^Cys^ (PDB ID: 1U0B) using PyMOL. (**B**) Schematic diagram of the screening approach. NMC-A reporter protein (PDB ID: 1BUE) is visualized using PyMOL. C70Am: Cys70Amber codon; C240Am: Cys240Amber codon. (**C**) Ampicillin resistance verification in obtained mutant and control groups. Bacterial suspensions adjusted to a consistent OD_600_ of 1 were applied onto plates with varying levels of ampicillin. Fluorescence observation (**D**) and fluorescence quantification (**E**) of the mutant and control groups. Quantitative analysis is presented as normalized fluorescence values. “Negative control” refers to no tRNA, and “positive control” indicates the absence of an amber codon in the EGFP reporter. The tRNA^Cys^_CUA_ and M1-1 are both controlled under the LeuQ promoter. Data represent the mean ± standard deviation (SD) (n = 3). ***P < 0.001; ns, not significant.

It was hypothesized that the evolved variant M1-1 might not be efficiently charged by endogenous aaRS and simultaneously by M38 due to modifications to the acceptor arm and the A58. Consequently, we subjected M38 to further directed evolution to augment its catalytic efficiency and specificity toward M1-1. Fig. 3A depicts selected residues proximate to the initial three base pairs of tRNA^Cys^. Site-directed mutagenesis employing NNK degeneracy was performed at these positions. The resultant library was ligated into the pRSF-M1-1 vector, and its sequence diversity was verified through sequencing. To increase the screening intensity, we introduced two amber codons into the active site of the NMC-A reporter. Successful Cys incorporation at these sites imparted functional activity to the NMC-A reporter [10] (Fig. 3B). We transformed the library plasmids into *E. coli* C321.ΔA.exp-pCDF-NMC-A-70/240TAG cells and conducted ampicillin resistance screening. A variant exhibiting a high level of resistance indicates greater catalytic efficiency for M1-1. Following multiple rounds of stringent selection, several superior variants were identified (Fig. S2). Sequencing revealed that these variants contained the same Gln301Ile mutation along with additional mutations. Therefore, we have designated all these variants with the same name: M38-18. We re-transformed this variant and re-tested its ampicillin resistance. The ampicillin resistance profiles for control cohorts and the M38-18 variant are provided in Fig. 3C. The result revealed that, compared to CysRS and variant M38, the engineered variant M38-18 imparted notably enhanced ampicillin resistance to M1-1, indicating greater catalytic efficiency toward M1-1.

To further validate the results obtained above, we employed EGFP as a reporter protein and introduced an amber codon at a permissive site within it. Fluorescence detection of colonies and bacterial suspensions revealed that when tRNA^Cys^_CUA_ was replaced with variant M1-1, the fluorescence intensity dropped significantly, becoming indistinguishable from background levels. This suggests that M1-1's cross-reactivity with all native aaRSs was substantially reduced, providing additional evidence that it is a more orthogonal tRNA. Based on M1-1, overexpression of CysRS and M38 failed to restore fluorescence; conversely, the introduction of M38-18 markedly enhanced fluorescence intensity, reaching levels almost equal to those of wild-type EGFP, once again demonstrating the specific high catalytic efficiency of the variant M38-18 for M1-1 (Fig. 3D). Quantitative fluorescence measurements are presented in Fig. 3E. The results showed the M1-1/M38-18 pair exhibited high decoding efficiency for the amber codon, with expression levels approaching those of the EGFP reporter without a TAG codon.

### 3.4 Characterization of the M1-1/M38-18 pair using GPx1 mutant as a reporter

**Fig. 4:**
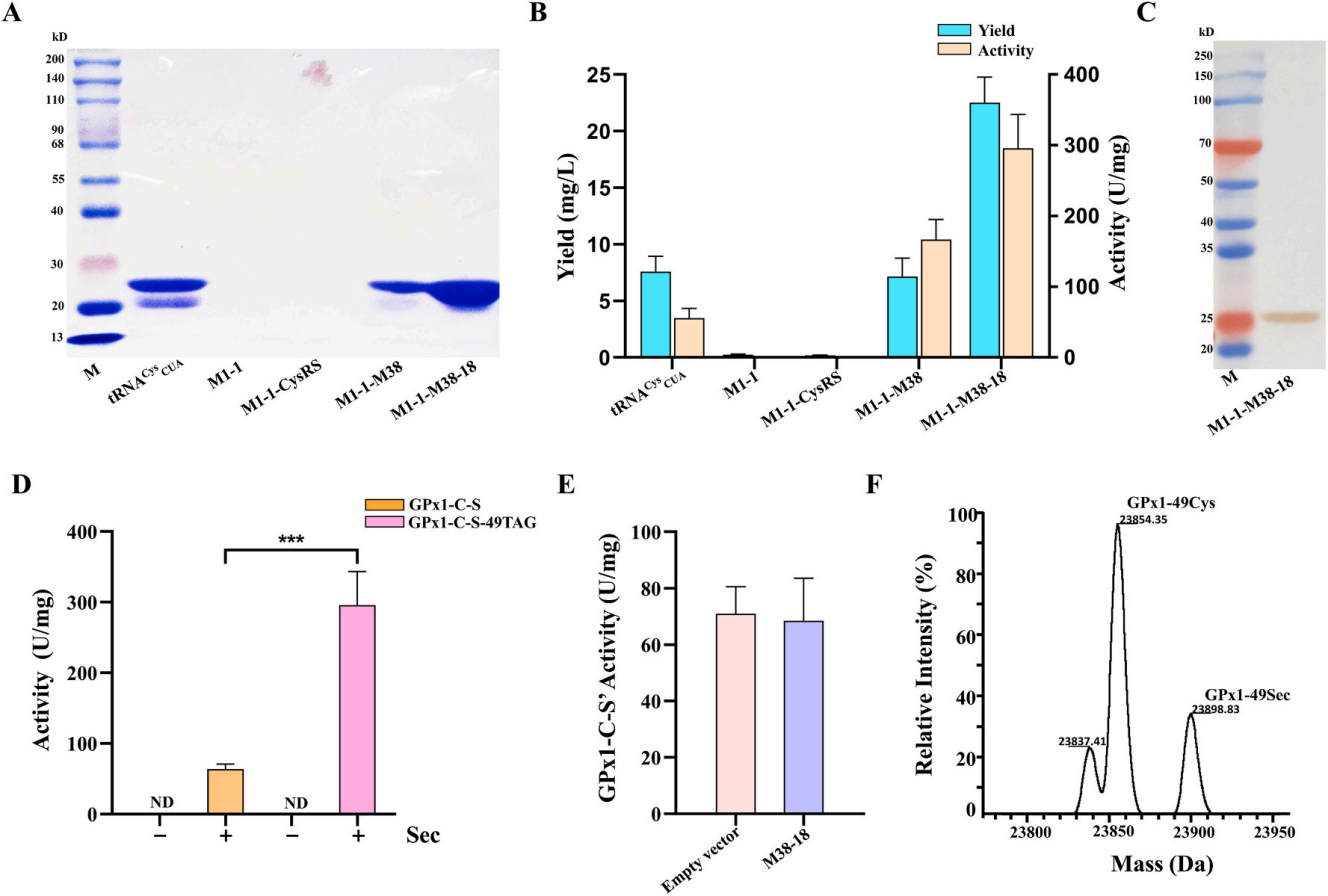
Functional validation of the M38-18/M1-1 pair based on selenoprotein GPx1-C-S-49TAG expression. (**A**) SDS-PAGE analysis of GPx1-C-S-49TAG expressed by the M38-18/M1-1 pair group and control groups. (**B**) Total protein yield and activity analysis of GPx1-C-S-49TAG expressed by the M38-18/M1-1 pair group and control groups. (**C**) Western blotting analysis of GPx1-C-S-49TAG expressed by the M38-18/M1-1 pair. (**D**) Activity assay of the GPx1 mutant expressed via normal decoding of the cysteine codon (GPx1-C-S group) or amber codon suppression using the M38-18/M1-1 pair (GPx1-C-S-49TAG group). Measurements were taken with and without the Sec separately. (**E**) Activity measurement of GPx1-C-S obtained by additionally introducing the M38-18 or by not. (**F**) Mass spectrometry detection of Sec in GPx1-C-S-49TAG expressed by the M38-18/M1-1 pair. M: Marker. ND: “Not Detected”. Empty vector: pRSF-DuetI. Data represent the mean ± standard deviation (SD) (n = 3); ***P < 0.001.

In order to further elucidate the availability of M1-1 and M38-18, we selected GPx1-C-S-49TAG, a genetically modified selenoprotein featuring serine substitutions for all cysteine residues and an amber codon at position 49, as a reporter system to examine protein expression. As depicted in Fig. 4A, SDS-PAGE analysis of purified GPx1-C-S-49TAG unambiguously revealed a noticeable reduction in protein expression when tRNA^Cys^_CUA_ was switched to M1-1, irrespective of the overexpression of wild-type CysRS. This further substantiates that the M1-1 variant is scarcely recognized by aaRS within *E. coli*. Conversely, co-expression of M38 and M1-1 enhanced protein expression, with an even more pronounced improvement observed in the combination of M38-18 and M1-1. The quantitative assessment of expression levels is presented in Fig. 4B. The total protein yield of GPx1-C-S-49TAG protein expressed using M38-18 and M1-1 is comparable in magnitude to that of the previous Cys auxotrophic system [21]. To confirm the expression, Western blotting was performed on the expression products generated by M1-1 and M38-18, as suggested in Fig. 4C. The above results again demonstrate the developed M38-18/M1-1 system exhibits excellent amber codon read-through efficiency.

Given the structural similarity between Sec and Cys and the ability of wild-type CysRS to incorporate Sec, we tested the competence of the M38-18/M1-1 pair in Sec incorporation. As shown in Fig. 4B, an enzymatic activity of approximately 290 U/mg was unexpectedly detected. To ascertain the activity attributable to Sec incorporation, we analyzed the activity of GPx1-C-S-49TAG and GPx1-C-S, both with and without Sec supplementation. The results in Fig. 4D revealed that only upon feeding with Sec could the activity emerge. Moreover, compared to the native CysRS and tRNA^Cys^_GCA_ pair, the M1-1 and M38-18 pair demonstrated a substantial increase in Sec incorporation, exhibiting a fourfold elevation in the activity of the GPx1 mutant. Combined with the GPx1 activity measured in the tRNA^Cys^_CUA_ control group shown in Fig. 4B, these results lead to an interesting conclusion: the evolved M38-18 may exhibit a greater preference for Sec compared to CysRS.

Since M38-18 is derived from CysRS and retains the majority of its amino acid sequence, including the catalytic site, it is expected to primarily charge tRNA^Cys^_GCA_ if cross-reaction occurs in *E. coli*. Consequently, we explored whether the overexpression of M38-18 could effectively outcompete endogenous CysRS, thereby enhancing the activity of GPx1-C-S. Fig. 4E showed no significant difference in activity in the presence or absence of M38-18, suggesting that M38-18 exhibits minimal recognition of endogenous tRNA^Cys^_GCA_ or fails to effectively compete with native CysRS. These findings indicate that when native GPx1 is expressed, the CysRS/tRNA^Cys^_GCA_ pair and the M38-18/M1-1 pair function independently. This independent action ensures optimal Sec insertion at the amber codon while minimizing Sec insertion at the cysteine codons. To validate Sec incorporation, the purified GPx1-C-S-49TAG produced by M1-1 and M38-18 was subjected to electrospray ionization mass spectrometry (ESI-MS) for intact mass analysis, which further confirmed Sec incorporation as shown in Fig. 4F. Semi-quantitative analysis of intact MS peak intensities indicated an approximate 75% Cys and 25% Sec incorporation at position 49, which represents an important aspect for future optimization of the system.

### 3.5 Expression and characterization of the natural GPx1 using the pair of M1-1 and M38-18

**Fig. 5:**
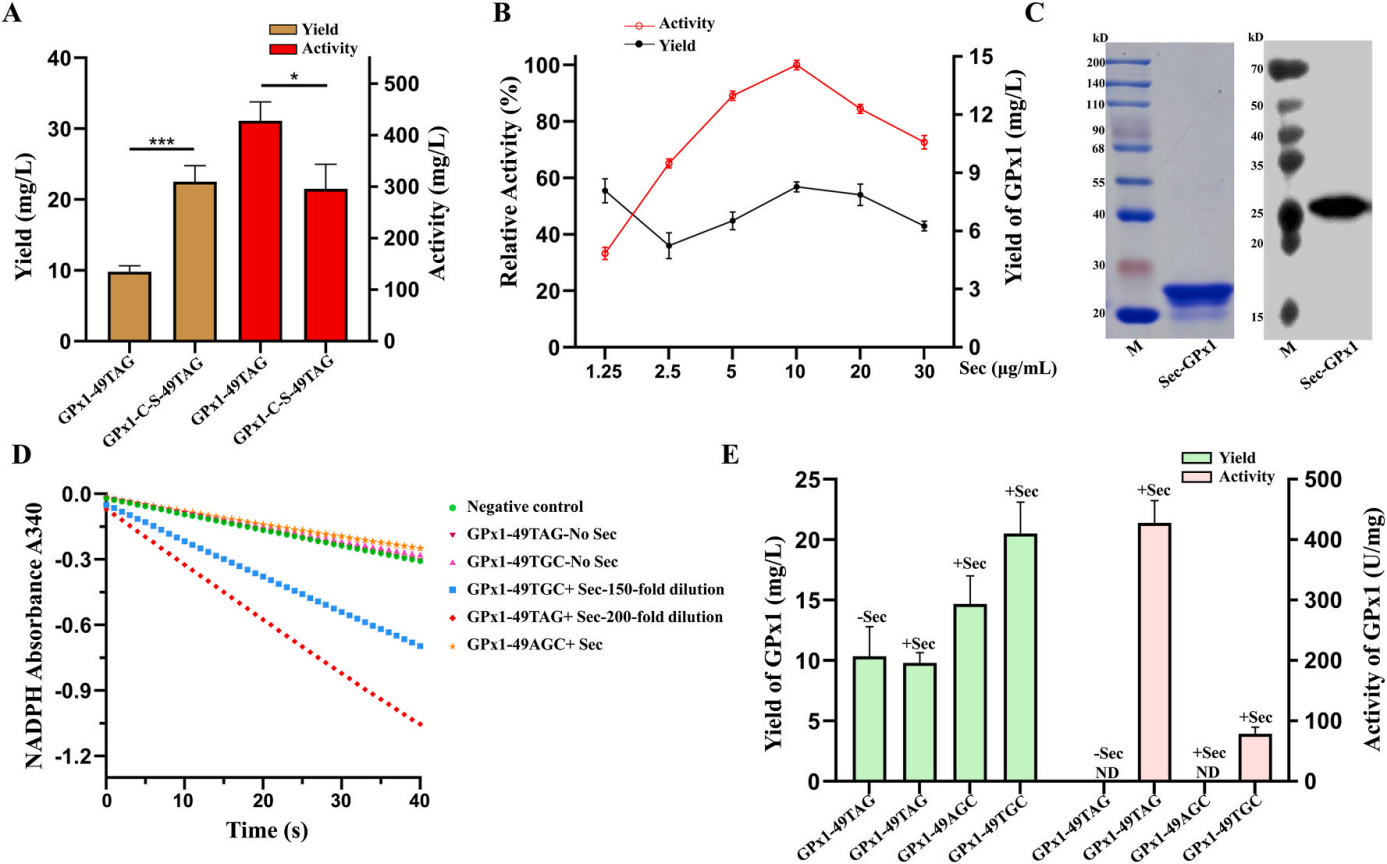
Expression and characterization of natural GPx1 using the M1-1/M38-18 pair. (**A**) Total protein yield and activity of GPx1-49TAG (natural GPx1) expressed by M1-1 and M38-18. (**B**) Total protein yield and activity of GPx1-49TAG expressed by M1-1 and M38-18 using different concentrations of Sec. Activity is represented as a relative value, with the maximum set to 100%. (**C**) SDS-PAGE and Western blotting analyses of GPx1-49TAG expressed by the M1-1/M38-18 pair. (**D**) Measurement of NADPH absorbance at A340 over time using H_2_O_2_ as a substrate. Negative control: Blank solution only. (**E**) Total protein yield and activity measurements of GPx1 with position 49 changed to TAG, TGC, and AGC codons, respectively. ND: “Not Detected”. Data represent the mean ± SD (n = 3); *P < 0.05, ***P < 0.001.

Considering that the GPx1 mutant is likely suboptimal for achieving highly active GPx1 and hinders research into the structure and function of natural selenoproteins, we pursued the expression of native GPx1 utilizing the M1-1 and M38-18 paired system. The results indicate a reduced yield accompanied by an increased activity of GPx1 in comparison to the GPx1 mutant (Fig. 5A). An enzyme activity of approximately 400 U/mg exceeds those previously documented in the literature [13] and reaches approximately half the level of purified natural GPx1 [29]. To improve the efficiency of Sec incorporation and to minimize the toxicity to *E. coli,* we examined the effect of varying Sec concentration. As illustrated in Fig. 5B, a Sec concentration of 10 μg/mL proved adequate for generating highly active GPx1 while maintaining relatively stable expression levels. Disruption of *CysE* [30], an essential gene in Cys biosynthesis, didn't enhance enzyme activity and instead led to impaired expression and observable growth deficits in Luria-Bertani medium (data not shown). SDS-PAGE and Western blot analysis yielded a distinct band at approximately 25 kDa, consistent with the theoretical molecular weight of 24 kDa, thereby confirming the successful expression of GPx1 (Fig. 5C). In addition, denaturing non-reducing electrophoresis analysis showed that the expressed GPx1 exists as a tetramer, consistent with its natural state (Fig. S3).

Subsequently, we aimed to confirm that the observed enzymatic activity was directly attributable to the targeted insertion of Sec into the GPx1 active site. Using H_2_O_2_ as the reaction substrate, time-course measurements of NADPH absorbance demonstrated that, in the absence of Sec supplementation during protein expression, NADPH oxidation proceeded slowly, in accordance with the changes observed in the negative control group. Among the expression products obtained following Sec supplementation, the GPx1-49AGC group continued to exhibit slow changes, while the rate of NADPH depletion in the GPx1-49TGC and GPx1-49TAG groups accelerated significantly. Moreover, the consumption rate in the GPx1-49TAG group remained superior to that in the GPx1-49TGC group (Fig. 5D). Calculations of total yield and activity suggest that, regardless of whether Sec is added, the yield in the GPx1-49TAG group remains almost unchanged. Consistent with prior observations, GPx1-49TAG exhibited a loss of activity in the absence of Sec. Analysis of the control group, GPx1-49AGC, demonstrates that non-specific substitution of cysteine residues with Sec at the cysteine sites does not confer enzymatic function. The activity observed in the GPx1-49TGC control group suggests that Sec integration into the cysteine site does occur, to a lesser extent than with M1-1/M38-18 translation systems (Fig. 5E).

**Fig. 6:**
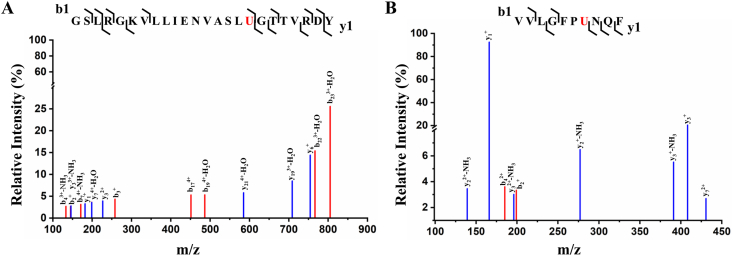
Mass spectrometry analysis of natural GPx1 expressed using the M1-1/M38-18 pair. (**A**) MS/MS spectrum of the quadruply charged precursor ion at *m*/*z* 653.82 ([M+4H]^4+^) corresponding to the peptide GSLRGKVLLIENVASLUGTTVRDY from GPx1. (**B**) MS/MS spectrum of the doubly charged precursor ion at *m*/*z* 586.25 ([M+2H]^2+^) corresponding to the peptide VVLGFPUNQF from GPx1.

The GPx1-49TAG sample expressed by our system was subjected to LC-MS/MS analysis for identification, which confirmed the successful incorporation of Sec at residue 49, as illustrated in Fig. 6A. Table S2 provides a detailed list of the molecular weights of all b-ions and y-ions with exact matches (i.e., fragment ions not resulting from neutral losses) highlighted in yellow. Additionally, non-specific Sec substitution at cysteine residues was identified, exemplified by the second cysteine residue of GPx1 (Fig. 6B). Relative quantitative analysis based on peak areas utilizing peptide segments of RGKVLLIENVASL*GTTVRDY and VVLGFP*NQF demonstrates that the Sec insertion efficiency of the target 49 residue is approximately twofold higher than that of the cysteine site of residue 78. Table S3 provides detailed information on the peak areas.

### 3.6 Expression of other natural selenoproteins using the M1-1/M38-18 pair

**Fig. 7:**
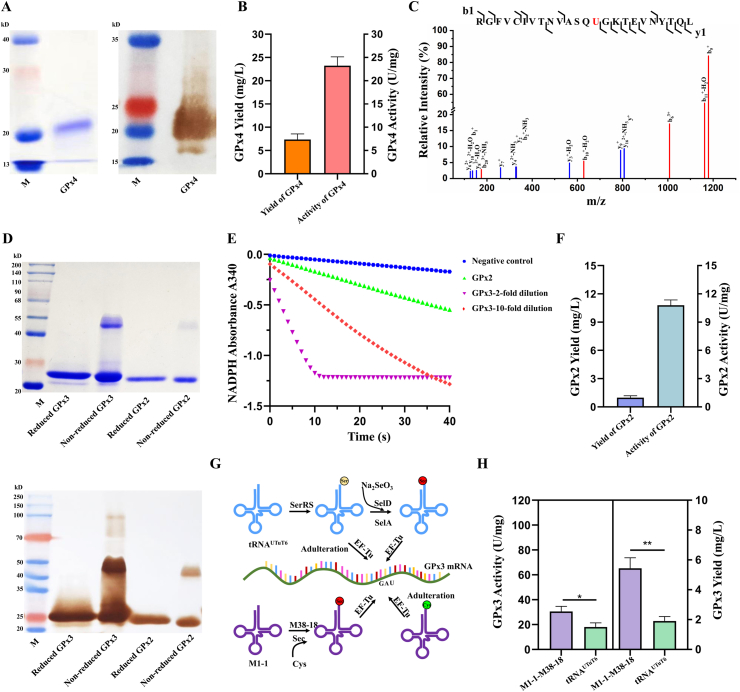
Expression of other GPxs using the M1-1/M38-18 system. (**A**) SDS-PAGE and Western blotting analyses of GPx4 expressed by the M1-1/M38-18 pair. (**B**) Total protein yield (mg/L) and activity (U/mg) of GPx4 expressed by the M1-1/M38-18 pair. (**C**) LC-MS/MS detection of Sec incorporation at position 46 in GPx4. (**D**) Determination of recombinant GPx2 and GPx3 expression by SDS-PAGE and Western blotting. “Non-reduced” denotes an experimental condition in which no reducing agents are added, and high-temperature denaturation is not performed. (**E**) Measurement of NADPH absorbance at A340 over time using H_2_O_2_ as a substrate. Negative control: Blank solution only. (**F**) Total protein yield (mg/L) and activity (U/mg) of GPx2 expressed by the M1-1/M38-18 pair. (**G**) Schematic diagram illustrating the two systems' implementation of site-specific Sec incorporation. (**H**) Comparison of GPx3 total protein yield and enzyme activity obtained using the UTuT6 system and M1-1/M38-18 system. Data represent the mean ± SD (n = 3); *P < 0.05, **P < 0.01.

To illustrate the versatility of the M1-1 and M38-18 pair, we selected an additional selenoprotein, GPx4, which is monomeric in natural conditions and functions to reduce multiple peroxides, including phospholipid hydroperoxides [31]. In addition, GPx4 is closely associated with the suppression of ferroptosis [32]; accordingly, investigations into the recombinant production of highly active GPx4 may hold significant promise for the treatment of ferroptosis-related diseases. As shown in Fig. 7A, using this M1-1/M38-18 pair, we successfully obtained an expressed product of approximately 20 kDa, which is in accordance with the theoretical molecular weight of GPx4 (21 kDa). After calculation, the enzyme activity was found to be approximately 23 U/mg (Fig. 7B), which is of the same order of magnitude as that of native GPx4 but moderately lower [29]. Mass spectrometric analysis of the GPx4 expression product revealed an incorporation of Sec at position 46 (Fig. 7C), confirming the applicability of the M1-1/M38-18 pair to other selenoproteins.

We proceeded to employ this pair for the recombinant expression of GPx2 and GPx3. GPx2 is a tetrameric selenoprotein mainly located in the gastrointestinal tract [33], while GPx3 is a tetrameric, glycosylated selenoprotein that is released into the plasma [34], where it carries out redox activities directly. SDS-PAGE and Western blot analyses of the expressed products are shown in Fig. 7D. Notably, under reducing conditions, both GPx2 and GPx3 display a single band around 25 kDa, indicating successful protein expression. However, under non-reducing conditions, both proteins migrate as multimers, which contrasts with previous reports [13,35]. The absence of a tetrameric band for GPx2 in the Western blot may be due to the denaturing effect of SDS, causing the protein to predominantly form dimers or even monomers.

Using H_2_O_2_ as the substrate, we measured the rate of NADPH depletion in the redox reactions catalyzed by GPx2 and GPx3, respectively (Fig. 7E). Compared to the control group, both accelerated NADPH oxidation, with GPx3 demonstrating a more pronounced effect. These findings indicate that the successful incorporation of Sec into the active site confers enzymatic activity and that GPx3 exhibits greater catalytic efficiency. The determined activity of the purified GPx2 enzyme is approximately 11 U/mg (Fig. 7F), a value comparable to that previously reported for recombinant GPx2 [36]. To further illustrate the advantages of our system, we conducted an additional replicate experiment expressing GPx3 via the UTuT6 system. As anticipated, beyond the introduction of Sec by both systems, the UTuT6 system unintentionally introduces Ser at the TAG codon, whereas our system introduces the undesirable Cys (Fig. 7G). Whenever Sec is exchanged for Cys or Ser in a GPx, the specific activity drops by two to three orders of magnitude or even more [37]. Consequently, enzyme activity specifically reflects the level of Sec incorporation. As depicted in Fig. 7H, GPx3 expressed via the UTuT6 system exhibits an activity of approximately 20 U/mg, which is broadly consistent with previously reported values [35,38]. In contrast, GPx3 expressed using our system demonstrates both a higher yield and moderate enhanced enzymatic activity. Considering the successful recombinant expression of diverse active GPx isoforms, we contend that this system harbors substantial potential for the expression of recombinant selenoproteins.

## 4 Discussion

Reactive oxygen species (ROS), inherently produced during physiological processes, are oxygenated compounds vital in small quantities for cellular signaling [39]. However, their overproduction induces oxidative stress, a condition linked to numerous pathologies. GPx, a class of selenoprotein enzymes with antioxidant properties, is essential for maintaining cellular redox homeostasis [40]. The complex recoding mechanism required for the specific incorporation of Sec into selenoproteins presents a significant hurdle in their development as biopharmaceutical agents.

Over the past two decades, genetic code expansion (GCE) technology has enabled the functionalization of proteins with non-canonical amino acids, imparting diverse physicochemical characteristics [41]. Our research endeavors focused on engineering a more orthogonal tRNA and aminoacyl-tRNA synthetase (aaRS) pair for Sec incorporation. Rather than selecting components from disparate biological origins, we pursued the directed evolution of *E. coli* endogenous CysRS and tRNA^Cys^. This work not only advances selenoprotein biosynthesis but also contributes to synthetic biology design and enhances our understanding of aaRS structure-function relationships.

We initiated this study by constructing an amber suppressor variant of tRNA^Cys^, specifically tRNA^Cys^_CUA_. We hypothesized that CysRS would not recognize tRNA^Cys^_CUA_, given that the anticodon, in conjunction with the discriminating base U73, is a primary determinant for synthetase interaction. A GCA to CUA mutation in the anticodon can disrupt critical interactions between CysRS and tRNA^Cys^ (Fig. 1B). For instance, the G34C substitution in the anticodon may switch 6-O, a hydrogen bond acceptor for Arg427, to 4-NH_2_, which is a hydrogen bond donor. The G34 residue is of critical importance, as its substitution diminishes aminoacylation efficiency by over 10^3^-fold [26,42]. Consequently, we targeted four residues within the anticodon-binding domain of CysRS for mutagenesis. Through iterative screening and validation, we identified an M38 mutant exhibiting superior performance (Fig. 1F and G). Comparable outcomes could be obtained from protein expression experiments.

**Fig. 8:**
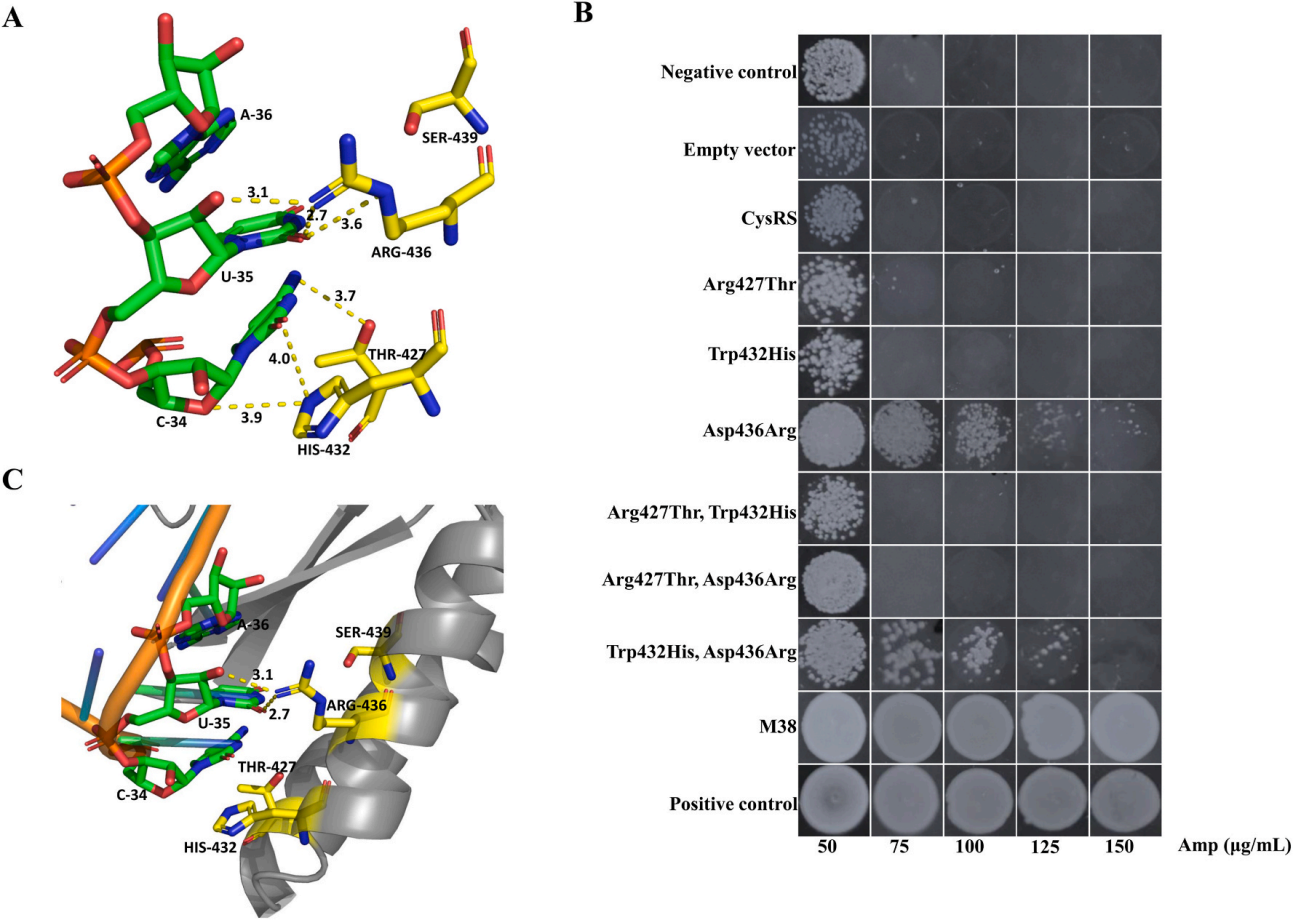
Analysis of the contribution of each amino acid in the M38 variant. The structure of M38 and tRNA^Cys^_CUA_ was generated by HDOCK [43] and displayed by PyMOL. (**A**) Distance of amino acids to potentially accessible nucleotides. Distances within 4 Å are marked with a yellow line. (**B**) Ampicillin resistance verification of constructed variants and control groups. Negative control: NMC-A-TAG; Empty vector group: pACYC-184 vector; Positive control: NMC-A without an amber codon. All experimental groups, except for the negative and positive control groups, were transfected with tRNA^Cys^_CUA_. (**C**) Hydrogen bond interaction between Arg436 and U35.

Our primary interest lies in discerning how the M38 variant manifests a pronounced affinity for tRNA^Cys^_CUA_ compared to the wild-type CysRS and the D436S mutant. The latter was generated in vitro via site-directed mutagenesis, predicated on a rational analysis of hydrogen bond donors and acceptors [25]. To this end, we systematically analyzed the contribution of each residue individually, with the exception of Ser439 due to its distant location. Fig. 8A illustrates the distances measured within a 4 Å proximity. Single and combined mutations were introduced at the other three sites within CysRS, and the ampicillin resistance of these variants was assessed. The results indicate that, relative to CysRS, the Asp436Arg variant alone enhances ampicillin resistance, albeit to a lesser extent than the M38 variant (Fig. 8B). These findings lead us to infer that the Asp436Arg mutation plays a pivotal role, whereas the other mutated residues likely contribute synergistically through the formation of interactive networks. The efficacy of the Arg436 mutation can likely be attributed to its capacity to form two critical hydrogen bonds with U35 of tRNA^Cys^_CUA_ (Fig. 8C).

Despite alterations to the anticodon, the tRNA^Cys^_CUA_ seemed to remain capable of being charged by endogenous CysRS (Fig. 1D and I). This may be due to the retention of U73, one of only two tRNAs in *E. coli* featuring a U at position 73, which is presumed to be significant for CysRS recognition [24]. Subsequently, we introduced mutations into the acceptor stem, an element considered to exert a substantial influence on aminoacylation specificity [44]. Adhering to the principle of enhancing orthogonality without incorporating additional tRNA identity elements, we designed and generated several variants and assessed their functionality using a more potent promoter (Fig. 2C). Antibiotic resistance testing revealed that the M1-1 variant exhibited significantly enhanced orthogonality (Fig. 2E). The M1-1 variant incorporates not only the initial three base pair mutations in the acceptor arm, derived from the M1 variant, but also an additional unexpected A58G mutation within the T loop, which we hypothesize can modulate the tRNA's tertiary structure. Typically, the invariant 58A engages in a reverse Hoogsteen pairing with the invariant 54T, maintaining stacking interactions within the T loop [45]. Further investigation is warranted to fully elucidate the functional role of the A58G mutation in M1-1.

Building upon the more orthogonal M1-1, we further evolved the M38 variant by randomizing residues proximal to the initial three base pairs. The resulting M38-18 mutant exhibited exceptional characteristics at the reporter level, including EGFP (Fig. 3E) and NMC-A (Fig. 3C), as well as in protein expression assays (Fig. 4A). To better elucidate the affinity between M38-18 and M1-1, we conducted molecular dynamics simulations of proteins and their corresponding ligands using GROMACS 2025, following their preparation via HDOCK docking. The binding free energy (ΔG_bind_) was estimated using the MM/PBSA method via the MMPBSA.py module in AmberTools [46]. Compared to the binding free energy of M1-1 and CysRS, a lower negative value was observed for M38-18 and M1-1, suggesting stronger interactions and higher affinity, as documented in Table S4. Table S4 provides a detailed breakdown of the binding energy components (including van der Waals, electrostatic, and solvation energies) for both systems.

Encouragingly, M38-18 demonstrates an augmented Sec incorporation rate relative to wild-type CysRS, as indicated by activity measurements of protein expressed in the presence of Sec (Fig. 4D). This difference may arise from mutations within the aaRS anticodon binding site and other regions, which modulate the amino acid binding site's tolerance for Sec through structural communication. It is worth noting that this does not necessarily indicate a preference of M38-18 for the substrate Sec over Cys, as we have yet to successfully evolve amino acid-binding sites of M38-18. Since both are substrates, the incorporation of Sec can be maximized by using an appropriate ratio. Regarding activity, a high insertion rate is already achievable at 10 μg/mL of Sec without significant toxicity. Compared to wild-type CysRS, M38-18 demonstrates an increased Sec/Cys affinity ratio, leading to a marked enhancement in activity under identical expression conditions.

The expression of native GPx1 using our M1-1/M38-18 system exhibits greater competitiveness compared to the GPx1 mutant lacking Cys residues in terms of activity, despite a reduction in yield (Fig. 5A). The total yield of GPx1 obtained in this work is approximately 10–20 mg/L with a Sec insertion rate of approximately 25%. The actual selenoprotein yield of 2.5–5 mg/L is on the same order of magnitude as that of the allo-UTu2D system (2-3 mg/L with 80% Sec incorporation, 1.6–2.4 mg/L effective selenoprotein) [11], but lower than that of the system utilizing the natural Sec recoding mechanism with SECIS (20 mg/L of thioredoxin reductases with 95% Sec incorporation, 19 mg/L effective selenoprotein) [47]. Furthermore, the enzymatic activity of GPx1 exceeds that of GPx1 expressed by the UTuT6 system (∼325 U/mg) and the UTuX system (∼300 U/mg) [13], yet remains inferior to that of GPx1 expressed by the allo-UTu2D system (>500 U/mg) [11].

Given the presence of five Cys residues in native GPx1, non-specific Cys-to-Sec substitution is foreseeable. Promiscuous insertions primarily stem from two sources: direct charging by endogenous CysRS and hydrolysis of Sec-tRNA, leading to free Sec that facilitates CysRS-mediated Sec misincorporation at Cys codons [11]. The latter means that selenoproteins expressed using any selenium source are likely to have Sec incorporated at the cysteine sites. Thus, the selenoproteins produced in our system exist as heterogeneous mixtures. In addition to the competitive insertion of Cys/Sec at the target site, Sec may also be incorporated at positions normally occupied by Cys. It is well established that the active site of GPx1 contains Sec, and its unique, conserved catalytic tetrad structure composed of Sec49, Gln84, Trp162, and Asn163 is critical for function [48]. Within the selenoprotein GPx family, substitution of Sec with its sulfur-containing homolog reduces enzymatic activity by two to three orders of magnitude [37]. Such a reduction is detrimental to enzymatic function and may render the activity undetectable. Consequently, incorporation of Cys at the target TAG site diminishes enzyme activity (Fig. 5D), and the proportion of such incorporation directly modulates overall activity. In contrast, incorporation of Sec at Cys sites may compromise enzymatic activity, particularly at high insertion rates [21], and does not confer enzymatic function (Fig. 5E). In comparison with GPx1-49TGC, GPx1-49TAG expressed using our M1-1/M38-18 system exhibits an identical Sec incorporation profile yet displays markedly elevated activity, further indicating that the M1-1/M38-18 pair and the tRNA^Cys^_GCA_/CysRS pair differ in their capacity to incorporate Sec at equivalent Sec concentrations. Notably, both the enzymatic activity and the purity of selenoproteins expressed by our system can be modulated by varying the supplemental Sec concentration. Consequently, optimal enzymatic activity reflects a higher Sec occupancy at the TAG site coupled with lower Sec incorporation at TGC/TGT sites. Although the expressed selenoprotein exhibits certain purity concerns, the overall enzymatic catalytic function remains unaffected.

We have tried to find a CysRS variant with improved affinity for Sec to minimize Sec consumption and reduce substitution at Cys codons. Because of the similar chemical properties of Sec and Cys and the scarcity of reporters capable of efficiently distinguishing between them for screening purposes, we attempted to screen desired mutants by employing positive and negative screening strategies with and without Sec, targeting the amino acid binding domain of M38-18. However, modifying the substrate specificity of CysRS proved to be a significant challenge since Cys and Sec differ by only a single atom, and we were unsuccessful. Following this, we investigated whether the endogenous CysRS-mediated charging of Sec could be attenuated. After the discovery of a Sec-rejecting CysRS in the selenium accumulator *Astragalus bisulcatus*, it has been proposed that a CysRS mutant can enhance selenium tolerance by lowering Sec misincorporation [49]. Therefore, we constructed a mutant strain harboring an H235N mutation in the *cysS* gene, which encodes CysRS, and measured the activity of selenoproteins expressed in this strain. As illustrated in Fig. S4, the H235N mutation in endogenous CysRS did not result in decreased enzymatic activity of GPx1-G-S, indicating that blocking Sec insertion using CysRS-H235N is limited. This discrepancy in results may be attributed to differences between using sodium selenite and the direct application of a high Sec concentration.

Nevertheless, we have successfully established an efficient amber suppression pair named M1-1/M38-18 and achieved the expression of GPx1 with high activity. Furthermore, our system has demonstrated efficacy in the recombinant expression of diverse active selenoproteins, including GPx2−4. System optimization for native selenoprotein expression may necessitate further exploration and in-depth investigation, such as developing a CysRS variant with greater specificity for Sec aided by the reporter gene hAGT-N137A, which has been reported to effectively distinguish Sec from Cys in knockout strains [50] and modifying the original Cys-tRNA^Cys^ synthesis pathway in *E. coli* to an indirect two-step process adapted from archaea [51], thereby potentially mitigating substrate conflicts with the M1-1/M38-18 pair.

In summary, we have, for the first time, engineered an amber suppression pair based on native CysRS and its cognate tRNA^Cys^ in *E. coli* and employed this pair to attain recombinant expressions of multiple active GPx isoforms. This innovative strategy provides a foundation for subsequent large-scale production and drug development initiatives.

## Data availability statement

Data will be made available on request.

## CRediT authorship contribution statement

**Shaopeng Niu:** Conceptualization, Data curation, Investigation, Methodology, Writing – original draft. **Jingyan Wei:** Conceptualization, Funding acquisition, Resources, Writing – review & editing. **Mengna Li:** Validation. **Borui Guo:** Supervision. **Qi Yan:** Investigation. **Yu Huang:** Writing – review & editing. **Zhenyan Jiang:** Methodology. **Jian Song:** Methodology.

## Declaration of competing interest

The authors declare the following financial interests/personal relationships which may be considered as potential competing interests: Jingyan Wei reports financial support was provided by National Natural Science Foundation of China. Jingyan Wei reports financial support was provided by Jilin Province Development and Reform Commission. If there are other authors, they declare that they have no known competing financial interests or personal relationships that could have appeared to influence the work reported in this paper.
