# Supplementary material for: Reassigning an amber codon to selenocysteine using a functional cysteine suppression pair in Escherichia coli
Source: Synth Syst Biotechnol. 2026 Jul 29;17:1–13. doi: 10.1016/j.synbio.2026.06.011 (PMC13449415; doi:10.1016/j.synbio.2026.06.011)
Supplement: Supplementary file 1 [file mmc1.docx]

**Supplemental data**

**Reassigning an amber codon to selenocysteine using a functional cysteine suppression pair in *Escherichia coli***

Shaopeng Niu ^a^, Jingyan Wei ^a,c,^*, Mengna Li ^a^, Borui Guo ^a^, Qi Yan ^a^, Yu Huang ^a^, Zhenyan Jiang ^a,^*, Jian Song ^b,^*

^a^ School of Pharmaceutical Science, Jilin University, Changchun, 130021, P. R. China.

^b^ School of microelectronics, Shanghai University, Shanghai, 201800, P. R. China.

^c^ Key Laboratory for Molecular Enzymology and Engineering of the Ministry of Education, Jilin University, Changchun, 130000, P. R. China.

* Corresponding author at: 1266 Fujin Road, Changchun 130021, PR China.

E-mail address: [weijy@jlu.edu.cn](mailto:weijy@jlu.edu.cn)

Correspondence may also be addressed to Jian Song. E-mail address: [jsong@shu.edu.cn](mailto:jsong@shu.edu.cn)

**Fig. S1. Testing for ampicillin resistance in constructed acceptor arm variants of tRNA^Cys^_CUA_**. Bacterial suspensions normalized to an OD_600_ of 1 were spotted onto ampicillin gradient plates at undiluted, 10-fold, and 100-fold dilutions. The undiluted sample is denoted in red, while the subsequent two wells correspond to the 10-fold and 100-fold dilutions, respectively. 1C2C3T: M1; 1C2C3T-58: M1-1; 1C2T3G: M2; 1C2A3G: M3; 1C2T3A: M4. All tRNA variants are under the control of the LeuQ promoter.

**Fig. S2. Screening for CysRS variants that exhibit enhanced catalytic activity toward M1-1 based on .** The left plate displays variants identified during initial screening for high-concentration ampicillin resistance, whereas the right plate presents the results of re-transformation of the isolated plasmids to confirm the ampicillin resistance of these variants. The rightmost panel presents the Sanger sequencing results for the identified CysRS variants.

**Fig. S3. Denaturing-reducing and denaturing-nonreducing SDS-PAGE analysis of recombinant GPx1 expressed by the M1-1/M38-18 pair.**

**Fig. S4. The construction of *E.coli* C321.ΔA.exp-*CysS*-H235N mutant and activity of GPx1-C-S expressed in this mutant strain.** *CysS*-H235N mutant strain was generated by λ-Red recombination. The results of resistance verification and DNA sequencing are displayed as above (left panel). GPx1-C-S is GPx1 mutant ,in which all serine residues are replaced with cysteine and the position 49 is TGC codon and decoded to cysteine by natural translation mechanism.

| Primer | sequence |
| --- | --- |
| F-tRNA^Cys^ | CCGGAATTCGGCGCGTTAACAAAGC |
| R-tRNA^Cys^ | CGCGGATCCTGGAGGCGCGTTCCGG |
| F-tRNA^Cys^-CUA | ATGTAGCGGATTCTAAATCCGTCTAGT |
| R-tRNA^Cys^-CUA | ACTAGACGGATTTAGAATCCGCTACAT |
| F-CysRS | GGAATTCCATATGCTAAAAATCTTCAATACTCTG |
| R-CysRS | CCGCTCGAGTTACTTACGACGCCA |
| F-CAT112TAG | AGTGAATACCACGACTAGTTCCGGCAGTTTCTA |
| R-CAT112TAG | TAGAAACTGCCGGAACTAGTCGTGGTATTCACT |
| F-CAT10TAG | AAAATCACTGGATATTAGACCGTTGATATATCC |
| R-CAT10TAG | GGATATATCAACGGTCTAATATCCAGTGATTTT |
| F-LeuQ-P | AGCTATTCCGTTACTCTAGAATGCATACCAAACCTG |
| R-LeuQ-P | CTTTGTTAACGCGCCATACCATCAATTCTTAA |
| F-tRNA^Cys^-M1-S1 | TTGATGGTATCCTGCGTTAACAA |
| R-tRNA^Cys^-M1-S1 | TTGTTAACGCAGGATACCATCAA |
| F-tRNA^Cys^-M1-S2 | CTCCGGAACGCAGGTCCAGGATCCT |
| R-tRNA^Cys^-M1-S2 | AGGATCCTGGACCTGCGTTCCGGAG |
| F-GPx1-C-S-49TAG | ATGTGGCGTCCCTCTAGGGCACCACGGTCCGG |
| R-GPx1-C-S-49TAG | CCGGACCGTGGTGCCCTAGAGGGACGCCACAT |
| F-GPx1-49AGC | ATGTGGCGTCCCTCAGCGGCACCACGGTCCGG |
| R-GPx1-49AGC | CCGGACCGTGGTGCCGCTGAGGGACGCCACAT |
| F-GPx1-49TGC | ATGTGGCGTCCCTCTGCGGCACCACGGTCCGG |
| R-GPx1-49TGC | CCGGACCGTGGTGCCGCAGAGGGACGCCACAT |
| F-CysRS-427/432/  436/439-NNK | CTGGATGCCNNKAAAGCGAAAGACNNKGCGGCGGCGNNKGCGGCGNNKGATCGTCTTA |
| R-CysRS-427/432/  436/439-NNK | TAAGACGATCMNNCGCCGCMNNCGCCGCCGCMNNGTCTTTCGCTTTMNNGGCATCCAG |
| F-CysRS-D436S | GACTGGGCGGCGGCGTCGGCGGCGCGTGATCGT |
| R-CysRS-D436S | ACGATCACGCGCCGCCGACGCCGCCGCCCAGTC |
| F-CysRS-158/166-NNK | GTGCTGTCGCGTNNKGATCTCGACCAGCTGCAGGCANNKGCGCGCGTTGACGTG |
| R-CysRS-158/166-NNK | CACGTCAACGCGCGCMNNTGCCTGCAGCTGGTCGAGATCMNNACGCGACAGCAC |
| F-CysRS-229/232-NNK | GGCGGCGGTTCANNKCTGATGNNKCCGCACCACGA |
| R-CysRS-229/232-NNK | TCGTGGTGCGGMNNCATCAGMNNTGAACCGCCGCC |
| F-CysRS-261NNK | CTCGGGGATGGTGNNKGTTGACCGCGA |
| R-CysRS-261NNK | TCGCGGTCAACMNNCACCATCCCCGAG |
| F-CysRS-301NNK | CTATCGCAGCNNKTTGAACTACA |
| R-CysRS-301NNK | TGTAGTTCAAMNNGCTGCGATAG |

**Table S1. Primary primer sequences used for plasmids construction.**

|  | **b+** | **b2+** | **b3+** | **b4+** |  | **y1+** | **y2+** | **y3+** | **y4+** |  |
| --- | --- | --- | --- | --- | --- | --- | --- | --- | --- | --- |
| **1** | **58.02874** | **29.51801** | **20.01443** | **15.26264** | **G** |  |  |  |  | **24** |
| **2** | **145.06077** | **73.03402** | **49.02511** | **37.02065** | **S** | **2555.30947** | **1278.15837** | **852.44134** | **639.58282** | **23** |
| **3** | **258.14483** | **129.57605** | **86.7198** | **65.29167** | **L** | **2468.27744** | **1234.64236** | **823.43066** | **617.82482** | **22** |
| **4** | **414.24594** | **207.62661** | **138.7535** | **104.31694** | **R** | **2355.19338** | **1178.10033** | **785.73598** | **589.5538** | **21** |
| **5** | **471.26741** | **236.13734** | **157.76065** | **118.57231** | **G** | **2199.09227** | **1100.04977** | **733.70227** | **550.52852** | **20** |
| **6** | **599.36237** | **300.18482** | **200.45897** | **150.59605** | **K** | **2142.0708** | **1071.53904** | **714.69512** | **536.27316** | **19** |
| **7** | **698.43078** | **349.71903** | **233.48178** | **175.36315** | **V** | **2013.97584** | **1007.49156** | **671.9968** | **504.24942** | **18** |
| **8** | **811.51485** | **406.26106** | **271.17647** | **203.63417** | **L** | **1914.90743** | **957.95735** | **638.97399** | **479.48231** | **17** |
| **9** | **924.59891** | **462.80309** | **308.87116** | **231.90519** | **L** | **1801.82336** | **901.41532** | **601.27931** | **451.2113** | **16** |
| **10** | **1037.68298** | **519.34513** | **346.56584** | **260.1762** | **I** | **1688.7393** | **844.87329** | **563.58462** | **422.94028** | **15** |
| **11** | **1166.72557** | **583.86642** | **389.58004** | **292.43685** | **E** | **1575.65523** | **788.33126** | **525.88993** | **394.66927** | **14** |
| **12** | **1280.7685** | **640.88789** | **427.59435** | **320.94758** | **N** | **1446.61264** | **723.80996** | **482.87573** | **362.40862** | **13** |
| **13** | **1379.83691** | **690.42209** | **460.61715** | **345.71469** | **V** | **1332.56971** | **666.7885** | **444.86142** | **333.89789** | **12** |
| **14** | **1450.87402** | **725.94065** | **484.29619** | **363.47396** | **A** | **1233.5013** | **617.25429** | **411.83862** | **309.13078** | **11** |
| **15** | **1537.90605** | **769.45666** | **513.30687** | **385.23197** | **S** | **1162.46419** | **581.73573** | **388.15958** | **291.3715** | **10** |
| **16** | **1650.99012** | **825.9987** | **551.00156** | **413.50299** | **L** | **1075.43216** | **538.21972** | **359.1489** | **269.6135** | **9** |
| **17** | **1801.94375** | **901.47551** | **601.31943** | **451.24140** | **U** | **962.34809** | **481.67769** | **321.45422** | **241.34248** | **8** |
| **18** | **1858.96522** | **929.98625** | **620.32659** | **465.49676** | **G** | **811.39446** | **406.20087** | **271.13634** | **203.60407** | **7** |
| **19** | **1960.01289** | **980.51009** | **654.00915** | **490.75868** | **T** | **754.37299** | **377.69014** | **252.12918** | **189.34871** | **6** |
| **20** | **2061.06057** | **1031.03392** | **687.69171** | **516.0206** | **T** | **653.32532** | **327.1663** | **218.44662** | **164.08679** | **5** |
| **21** | **2160.12899** | **108,056,813** | **720.71451** | **540.7877** | **V** | **552.27764** | **27,664,246** | **184.76406** | **138.82487** | **4** |
| **22** | **2316.2301** | **1158.61869** | **772.74822** | **579.81298** | **R** | **453.20922** | **227.10825** | **151.74126** | **114.05776** | **3** |
| **23** | **2431.25704** | **1216.13216** | **811.09053** | **608.56972** | **D** | **297.10811** | **149.05769** | **99.70756** | **75.03249** | **2** |
| **24** |  |  |  |  | **Y** | **182.08117** | **91.54422** | **61.36524** | **46.27575** | **1** |

**Table S2. The m/z values for ESI-MS/MS spectrum of GSLRGKVLLIENVASLUGTTVRDY.** Matched theoretical values are marked in highlighted yellow.

| Annotated Sequence | Modifications | MH+ [Da] | Theo. MH+ [Da] | EIC Area |
| --- | --- | --- | --- | --- |
| RGKVLLIENVASLcGTTVRDY | C14(Cys->Dha) | 2273.21976 | 2273.26121 | 7260885.5 |
| RGKVLLIENVASLcGTTVRDY | C14(Cys->Ala) | 2275.24161 | 2275.27686 | 2757993 |
| RGKVLLIENVASLcGTTVRDY | C14(Carbamidomethyl) | 2364.32682 | 2364.27039 | 1393058 |
| RGKVLLIENVASLcGTTVRDY | C14(Cys->Sec) | 2355.18949 | 2355.19338 | 235375.1875 |
| RGKVLLIENVASLCGTTVRDY |  | 2307.31345 | 2307.24893 | 973809.4375 |
| RGKVLLIENVASLcGTTVRDY | C14(Cys->Ser) | 2291.26909 | 2291.27177 | 708558.1875 |
|  |  |  |  |  |
| Annotated Sequence | Modifications | MH+ [Da] | Theo. MH+ [Da] | EIC Area |
| VVLGFPcNQF | C7(Carbamidomethyl) | 1180.595 | 1180.582 | 3069806.5 |
| VVLGFPcNQF | C7(Cys->Ser) | 1107.567 | 1107.583 | 441101.4375 |
| VVLGFPCNQF |  | 1123.584 | 1123.56 | 2127661.75 |
| VVLGFPcNQF | C7(Cys->Ala) | 1091.568 | 1091.588 | 839007.75 |
| Annotated Sequence | Modifications | MH+ [Da] | Theo. MH+ [Da] | EIC Area |
| VVLGFPcNQF | C7(Cys->Sec) | 1171.495 | 1171.505 | 1532936.125 |
| VVLGFPcNQF | C7(Cys->Dha) | 1089.544 | 1089.573 | 1830629 |

**Table S3. Peptide peak areas and relative proportions for peptides with different amino acid modifications.** Red marks indicate confirmed Sec modifications.

| **Systems** | **M1-1-CysRS** | **M1-1-M38-18** |
| --- | --- | --- |
| △E_ele_ | 7361.84 | 3788.14 |
| △E_vdW_ | −270.93 | −268.98 |
| △G_GB_ | -7208.99 | −3930.39 |
| △G_SA_ | -13.81 | -0.47 |
| △G_pol_^a^ | 252.85 | −142.25 |
| △G_nonpol_^b^ | -284.74 | −269.45 |
| △G_total_^c^ | -131.89 | −411.70 |

^a^$\Delta G_{pol}=\Delta E_{ele}+\Delta G_{GB}$	^b^$\Delta G_{\mathrm{nonpol}}=\Delta E_{\mathrm{vdw}}+\Delta G_{\mathrm{SA}}$	^c^$\Delta G_{\mathrm{total}}=\Delta E_{\mathrm{ele}}+\Delta E_{\mathrm{vdw}}+\Delta G_{\mathrm{GB}}+\Delta G_{\mathrm{SA}}$

**Table S4. Binding free energy (kcal·mol-1).** The molecular dynamics (MD) simulations were performed using the GROMACS 2025.2 package, with the initial complex structure derived from M1-1-M38-18.pdb generated by HDOCK. The Amber ff99SB-ILDN force field was utilized for the protein, while the OL3 parameters were applied to the RNA component. Two structural Mg^2+^ ions, essential for RNA stability, were retained from the original coordinates and modeled using standard divalent ion parameters compatible with the Amber force field. Each system was solvated in a truncated octahedral box of TIP3P water with a minimum distance of 1.0 nm between the solute and box walls and neutralized with 0.15 M NaCl. After energy minimization using the steepest descent algorithm, the systems were equilibrated in the NVT ensemble for 100 ps at 310 K using the V-rescale thermostat, followed by 1 ns of NPT equilibration at 1.0 bar using the Parrinello-Rahman barostat. A 100 ns production MD run was executed for each system with a 2 fs time step. Long-range electrostatic interactions were treated with the Particle Mesh Ewald (PME) method, and all covalent bonds involving hydrogen atoms were constrained using the LINCS algorithm. For binding free energy analysis, the trajectories were stripped of periodic boundary conditions and solvent molecules, then converted to a format compatible with AmberTools. The binding free energy (ΔGbind) was estimated using the MM/PBSA method via the MMPBSA.py module in AmberTools, analyzing 200 snapshots from the final 20 ns of the production phase.
